# Pro-Inflammatory Profile of Adipokines in Obesity Contributes to Pathogenesis, Nutritional Disorders, and Cardiovascular Risk in Chronic Kidney Disease

**DOI:** 10.3390/nu14071457

**Published:** 2022-03-31

**Authors:** Sylwia Czaja-Stolc, Marta Potrykus, Marta Stankiewicz, Łukasz Kaska, Sylwia Małgorzewicz

**Affiliations:** 1 Department of Clinical Nutrition, Medical University of Gdansk, 80-211 Gdańsk, Poland; marta.stankiewicz@gumed.edu.pl (M.S.); sylwia.malgorzewicz@gumed.edu.pl (S.M.); 2 Department of General, Endocrine and Transplant Surgery, Medical University of Gdansk, 80-211 Gdańsk, Poland; martapotrykus@gumed.edu.pl (M.P.); lukasz.kaska@gumed.edu.pl (Ł.K.)

**Keywords:** obesity, adipokines, chronic kidney disease, malnutrition, cardiovascular risk

## Abstract

Obesity is a disease which leads to the development of many other disorders. Excessive accumulation of lipids in adipose tissue (AT) leads to metabolic changes, including hypertrophy of adipocytes, macrophage migration, changes in the composition of immune cells, and impaired secretion of adipokines. Adipokines are cytokines produced by AT and greatly influence human health. Obesity and the pro-inflammatory profile of adipokines lead to the development of chronic kidney disease (CKD) through different mechanisms. In obesity and adipokine profile, there are gender differences that characterize the male gender as more susceptible to metabolic disorders accompanying obesity, including impaired renal function. The relationship between impaired adipokine secretion and renal disease is two-sided. In the developed CKD, the concentration of adipokines in the serum is additionally disturbed due to their insufficient excretion by the excretory system caused by renal pathology. Increased levels of adipokines affect the nutritional status and cardiovascular risk (CVR) of patients with CKD. This article aims to systematize the current knowledge on the influence of obesity, AT, and adipokine secretion disorders on the pathogenesis of CKD and their influence on nutritional status and CVR in patients with CKD.

## 1. Introduction

The global epidemic of overweight and obesity has been constantly increasing. According to the World Health Organization (WHO), the prevalence of obesity in the world has almost tripled in the last fifty years. In 2016, over 650 million adults were obese [1]. Newly emerging publications indicate that the COVID-19 pandemic, and the accompanying lockdown, contributed to the exacerbation of obesity [2,3]. Excessive body weight contributes to the development of many diseases, such as cardiovascular diseases (CVD), diabetes, and chronic kidney disease (CKD). Treatment of obesity and diseases caused by it is associated with a significant financial burden for the health care system [4,5].

The mean global incidence of CKD is 13.4%, but many patients go undiagnosed [6]. Patients with CKD are characterized by higher mortality compared to the general population. The main cause of death in patients treated conservatively and undergoing dialysis are cardiovascular diseases [7]. The risk of developing CVD is 5–10 times higher in the CKD population compared to people with normal kidney function. Traditional risk factors such as lipid disturbances, diabetes, and hypertension do not explain this phenomenon [8]. Furthermore, untraditional risk factors such as chronic inflammation, hypoalbuminemia, or oxidative stress, included in protein-energy wasting (PEW), have a negative impact on survival [9].

Obesity increases the risk of developing CKD in many ways. For instance, it contributes to chronic inflammation, oxidative stress, impaired secretion of adipokines and lipotoxicity, which results in nephron damage [10,11]. The aim of the study is to review and summarize the literature on obesity, adipose tissue (AT), and adipokines, and their impact on the risk of developing CKD as well as the occurrence of nutritional disorders and cardiovascular risk (CVR) in the above-mentioned group of patients. To find articles matching the topic of the manuscript, PubMed and WorldWideScience databases were searched for keywords “obesity”, “adipokines”, “chronic kidney disease”, “leptin”, “adiponectin”, “zinc-α2-glycoprotein”, “adipose triglyceride lipase”, “cardiovascular risk”, and their various combinations. The bibliography of selected publications was also searched. It has been established that the articles considered in the search for information for the manuscript are to be published in the period from 2000 to 2022. All publications published earlier than 2000 and/or written in languages other than English and Polish were excluded.

## 2. Heterogeneity of Adipose Tissue

AT, as an endocrine organ, is a source of various substances including adipokines [12]. AT is a miscellaneous tissue which consists of adipocytes, preadipocytes, fibroblasts, immune cells, epithelial cells of the blood and lymphatic systems, and stem cells [13,14]. 

Depending on the structure and function of adipocytes, AT is divided into white, brown, and beige tissues. White adipose tissue (WAT) stores fatty acids as an energy source and consists of one large, centrally placed droplet of triglycerides (TG) and other cell organelles pushed to the cell periphery [15]. In turn, adipocytes included in brown adipose tissue (BAT) contain numerous smaller droplets of fatty acids and a high number of mitochondria. BAT is rich in the mitochondrial protein—uncoupling protein 1 (UCP1), which dissipates energy in the form of heat and thus increases energy expenditure. Unlike WAT, BAT reduces hyperlipidemia and facilitates weight loss [16,17]. It is possible to induce UCP1 expression in white adipocytes, a process known as adipocyte browning. The cells created in this way are beige adipocytes which, despite their different origins, have a similar structure and function to brown adipocytes. Browning of adipocytes induced by various factors, including exposure to cold, insulin, specific nutrients, or the presence of an excess of nutrients, has a positive effect on health [17,18].

Additionally, WAT can be classified based on its distribution in the body. Although it is found almost throughout the body, there are several places where it is most abundant. These include subcutaneous, pericardial, gonadial, and visceral adipose tissue (VAT) divided into mesenteric, omental, and retroperitoneal AT [19,20]. As already mentioned, AT is scattered throughout the body. This type of AT is called discrete tissue-associated adipose depots. They are small clusters that are closely related to other anatomical structures and perform functions specific to these structures [19]. 

## 3. Obesity-Induced Changes in Adipose Tissue

Obesity is characterized by an increase in and dysregulation of WAT, especially visceral fat, which is associated with altered adipokine secretion. When the threshold of adipocyte storage capacity for fatty acids is exceeded, the processes of hypertrophy and hyperplasia take place [17]. An increased size of fat cells (hypertrophy) and their increased number (hyperplasia) differ in the way they affect the organism. Hypertrophy is associated with metabolic disturbances, while hyperplasia shows protective activity [21]. Under conditions of positive energy balance, the size and number of adipocytes increases. Increased adipocytes secrete some hormones and cytokines that affect both pre-adipocyte recruitment and pre-adipocyte differentiation into adipocytes. In the case of a chronic positive energy balance, adipocytes become lipid-overloaded and reach a critical size, which leads to inhibition of cellular multiplication and an imbalance between the processes of hypertrophy and hyperplasia [22].

The hypertrophic adipocytes secrete pro-inflammatory cytokines such as tumor necrosis factor-α (TNF-α), interleukin 6 (IL-6), interleukin 8 (IL-8), and monocyte chemoattractant protein 1 (MCP-1) which, through the phosphorylation of insulin receptor substrate-1 (IRS-1), cause insulin resistance. The secretion of these cytokines also recruits macrophages and T-cells, which fuel inflammatory processes. Enlarged adipocytes also cause local tissue hypoxia, which results in the release of hypoxia-inducible factor (HIF-α), leading to inflammation and fibrosis of the AT. If the capacity of the adipocytes is exceeded, lipids start to accumulate in the ectopic tissues, which deteriorates the functioning of these organs and/or tissues [22].

**Figure 1:**
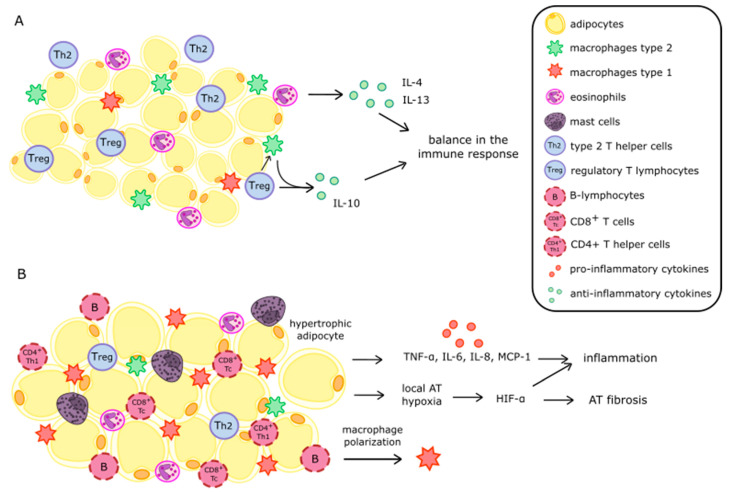
Comparison of adipose tissue of humans with normal body weight (**A**) and with obesity (**B**). Abbreviations: AT—adipose tissue; IL-4,-6,-8,-10,-13—interleukin -4,-6,-8,-10,-13; TNF-α—tumor necrosis factor α; MCP-1—monocyte chemoattractant protein-1; HIF-α—hypoxia-inducible factor α.

AT is characterized by the presence of various immune cells such as type 2 T-helper cells (Th2), regulatory T-lymphocytes (Treg), eosinophils and type 2 macrophages (M2 macrophages). Treg causes the secretion of anti-inflammatory interleukin 10 (IL-10) and promotes the formation of M2 macrophages which suppress inflammation through the secretion of IL-10. Eosinophils are also characterized by the secretion of anti-inflammatory cytokines interleukin 4 (IL-4) and interleukin 13 (IL-13). This composition of immune cells allows for homeostasis and anti-inflammatory effects [23]. Obesity causes disturbances in the composition of the immune system cells in AT. Hypertrophic adipocytes cause monocytes to migrate to AT and transform them into type 1 macrophages (M1 macrophages). The number of B lymphocytes also increases, which enhances the formation of M1 macrophages. A decrease in the number of eosinophils causes a fall in the secretion of anti-inflammatory cytokines—IL-4 and IL-13. In addition, obesity diminishes the amount of Treg in AT, which results in an increase in cytotoxic CD4^+^Th1 and CD8^+^Tc [23,24]. Figure 1 compares AT in people with obesity and normal body weight and the consequences of their metabolic activity.

An increased amount of WAT, through increased release of pro-inflammatory cytokines, reduces BAT activity by inhibiting the induction of UCP1. It is associated with a decreased energy expenditure and might be another reason for difficulties in losing and maintaining weight, and obesity [17,25].

## 4. Sexual Dimorphism in the Obesity-Induced Metabolic Profile

The central distribution of AT typical of men is correlated with a greater incidence of metabolic disorders than the gluteal–femoral distribution of AT. Additionally, a pear-type silhouette is associated with a reduced risk of metabolic disease and may have a protective effect against the consequences of obesity in both sexes [26].

Estrogens, which dominate in women, increase the level of leptin and other anorexic compounds such as cholecystokinin, brain-derived neurotrophic factor (BDNF), and apolipoprotein A-IV, and simultaneously reduce orexigenic substances, including ghrelin and melanin-concentrating hormone. The protective effect of estrogens against obesity is not only limited to reduced food consumption but also increased energy expenditure [27].

Gender not only affects the distribution and type of AT but also affects the type of metabolic activity of AT. Estrogens reduce inflammation in AT and make it more insulin-sensitive. Estrogens reduce the activity of HIF-α, which is responsible for fibrosis and inflammation, by promoting the transcription of the enzyme prolyl hydroxylase domain enzyme 3 (PHD3) [27].

In turn, testosterone inhibits the activity of lipoprotein lipase, which reduces the uptake of fatty acids in the abdominal fat tissue. Chronic overexposure to free fatty acids (FFAs) in the circulation increases hepatic glucose production and elevates insulin production. It leads to a decrease in tissue sensitivity to insulin. This phenomenon is one of the reasons why men are more susceptible to insulin resistance and metabolic syndrome [26].

The greater deposition of VAT in men, and therefore a greater risk of high blood pressure and CVD, may be due to higher testosterone levels in men than in women. In addition, in postmenopausal women, decreased levels of estrogens also lead to increased VAT and higher blood pressure [28].AT concentrated around the kidneys is called perirenal adipose tissue (PRAT) and is part of visceral white adipose tissue. The morphology of PRAT is gender-dependent. In males, PRAT has a greater volume and thickness than in females with the same waist circumference. Additionally, in women, the expression of UCP1 in PRAT is higher than in men [29]. 

In the murine model, it was observed that the presence of the Y chromosome inhibits the expression of UCP1 in BAT. However, Chen et al. suggest that it is not the presence of the Y chromosome that leads to the gender differences in AT, but the increased number of X chromosomes. The authors showed that having a set of two X chromosomes led to a higher body weight to fat mass ratio compared to one X chromosome regardless of the number of Y chromosomes [30]. 

In people with obesity, there is sexual dimorphism in the levels of adipokines and their correlation with metabolic disorders. Walicka et al. observed that women had higher levels of adiponectin, leptin, and visfatin. In women, there was a negative correlation between the levels of adiponectin and insulin and the homeostasis model assessment of insulin resistance (HOMA-IR) index. In turn, in men, there was a positive correlation between the level of leptin and the HOMA-IR index [31]. It has been suggested that the leptin-to-adipokine (Lep/Adpn) ratio is an indicator of metabolic disorders. In the study by Selthofer-Relatić et al., in women, this ratio positively correlated only with anthropometric measurements. In men, however, the higher the Lep/Adpn ratio, the worse the results of total cholesterol, low-density lipoprotein (LDL) fraction, and TG are. This suggests that differences in the secretion of adipokines in different sexes affect the neurohormonal activity of AT. This leads to differences in the metabolic profile [32].

Females, at least before menopause, are more protected against obesity and inflammatory changes in AT. They are also more likely to maintain the proper functioning of kidneys. Across all classes of CKD, men show more kidney damage and a faster progression to end-stage renal disease (ESRD) compared to premenopausal women. After menopause, this effect wears off [33]. 

## 5. Obesity and Chronic Kidney Disease

CKD is defined by the disturbance of the function or structure of the kidneys lasting more than three months. CKD is divided into five stages based on the value of the glomerular filtration rate (GFR) [34]. It is estimated that the mean global incidence of CKD is 13.4%, but many patients go undiagnosed and find out about the disease in the late stages of development [6]. 

**Figure 2:**
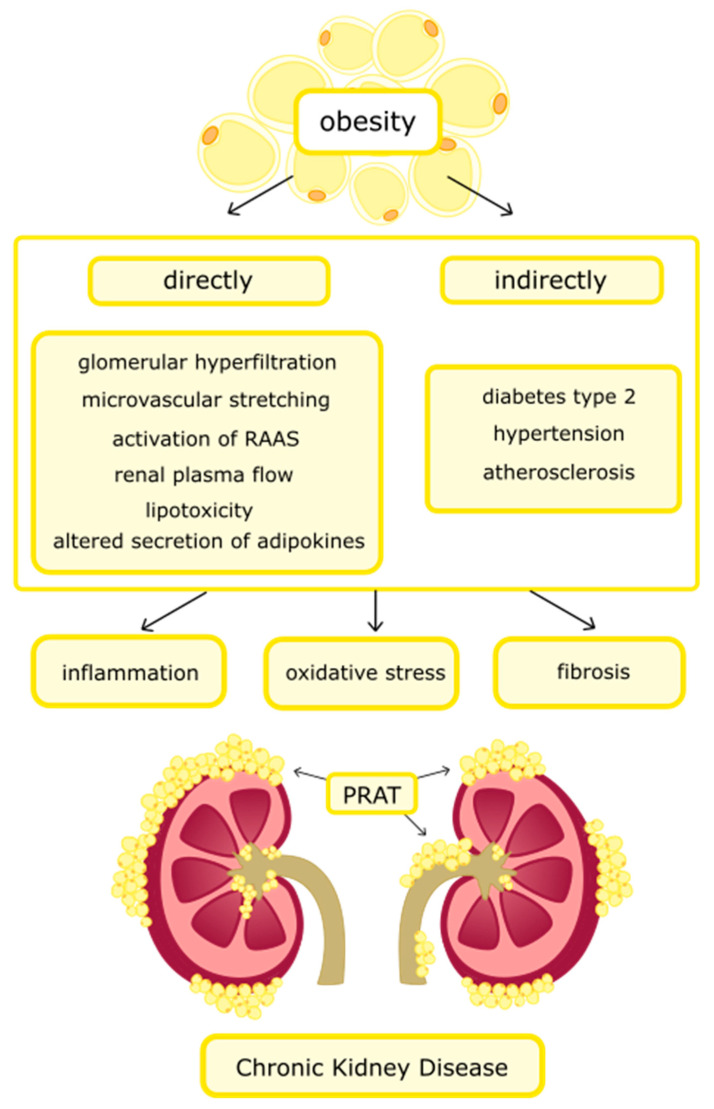
The direct and indirect impact of obesity on the development of chronic kidney disease. Abbreviations: RAAS—renin-angiotensin-aldosterone system; PRAT—perirenal adipose tissue.

Overweight and obesity contribute to the development of CKD among 15–30% of patients, but the mechanism of kidney damage is not fully understood [35]. Obesity increases the risk of developing CKD in a direct and indirect way, as is shown in Figure 2. The direct effect is associated with glomerular hyperfiltration (abnormally high GFR), increased renal plasma flow, microvascular stretching, renin–angiotensin–aldosterone system (RAAS) activation, altered secretion from AT, and lipotoxicity. All of these factors promote the formation of inflammation, oxidative stress, and fibrosis in the kidneys [11,36]. The pathophysiological mechanism described above leads to the development of obesity-related glomerulopathy (ORG) and is characterized by glomerulomegaly. If it is accompanied by glomerulosclerosis, it is called focal and segmental glomerulosclerosis (FSGS) [37]. The first symptom of ORG is usually microalbuminuria or clinically dominant proteinuria [38]. These disorders lead to progressive damage to nephrons and the development of CKD. The incidence of it is steadily increasing, however, due to relatively infrequent renal biopsy performed, exact statistics are not known [39,40]. Research is underway to discover new biomarkers of kidney damage. One of them is micro-RNA, which can be measured in the blood [41]. The indirect effect of CKD development is related to hypertension, atherosclerosis, and diabetes type 2 [35].

Hyperfiltration occurs as a result of dilatation of the afferent arteriole to the glomerulus, which is associated with an increase in reabsorption of glucose and sodium via sodium–glucose co-transporter–1 (SGLT1) and sodium–glucose co-transporter–2 (SGLT2), which leads to renal hypertrophy. The phenomenon manifested by an increase in the volume of glomerular tuft/capsule, tubular epithelium/lumen, and podocyte injury. These changes consequently lead to glomerulosclerosis [42,43]. A physiological glomerular hyperfiltration occurs after consuming a large amount of protein, sodium, and during pregnancy [44,45]. Glomerulomegaly is another renal pathology associated with obesity that occurs independently of microalbuminuria. These processes lead to impaired kidney function and complications associated with it [11,45]. 

Lipotoxicity is defined as the ectopic accumulation of lipids in organs other than AT. PRAT and accumulation of fatty acids in renal parenchyma cause the damage of tubulointerstitial tissue, proximal tubular epithelial cells, and endothelial cells [46]. Non-esterified fatty-acid (NEFA) accumulation leads to insulin resistance and apoptosis of podocytes which cannot regenerate [39,47,48]. Insulin resistance and hyperinsulinemia lead to activation of the sympathetic nervous system, which was confirmed by an increase in the concentration of catecholamines, and contributes to the occurrence of vasoconstriction [49]. Cumulation of NEFA in renal cells activates macrophages, which leads to transformation into foam cells [46]. Renal steatosis leads to the development of inflammation and fibrosis. Lipotoxicity contributes to decreased production of adenosine 5′-triphosphate (ATP) through inhibition of β-oxidation, thereby damaging the structure of the mitochondria [50]. PRAT compresses the kidneys, which causes an increase in hydrostatic pressure, reduces renal blood flow, and leads to CKD [51]. The influence of adipokines on the risk of developing CKD is described in the next section.

## 6. Adipokines and Their Influence on Pathogenesis, Nutritional Disorders, and Cardiovascular Risk in Chronic Kidney Disease

### 6.1. Leptin

Leptin is a plasma protein encoded by the obesity gene (ob) that was first described in 1994. It is an anorexigenic hormone that increases energy expenditure. In obesity, increased concentration of leptin occurs, simultaneously with leptin resistance [52]. Reduced leptin activity on the hypothalamus impairs appetite suppression [53].

Enlarged adipocytes are characterized by increased secretion of leptin. Thus, adipokine, by binding to leptin receptors in the central nervous system, causes increased activity of the sympathetic nervous system, which, consequently, can lead to obesity-related hypertension [34]. Hypertension is inextricably linked with kidney failure. Although hypertension is one of the greatest risk factors for the development of CKD, it is also a result of impaired renal function. This is manifested by the fact that with the progression of CKD, the incidence of hypertension increases [54].

Leptin increases the expression of the transforming growth factor-β 1 (TGF-β1) gene and other fibrotic factors, such as collagen IV and fibronectin, which stimulate the proliferation of mesangial cells in the kidneys [55]. Excessive production of these cells can lead to glomerulosclerosis through mesangial hypertrophy in the glomeruli, thickening of the glomerular basement membrane, and increased extracellular matrix [11]. In turn, glomerulosclerosis, by increasing the permeability of the glomerular barrier, contributes to proteinuria and impaired renal function [56].

Leptin-induced vascular endothelial dysfunction is another mechanism that links altered adipokine secretion in obesity with impaired renal function. Leptin stimulates the formation of reactive oxygen species (ROS) that impair the vascular response to acetylcholine, thereby initiating reduced bioavailability of nitric oxide and oxidative stress [57]. In addition, leptin modulates the immune system response. The hormone increases the number of T-helper cells and reduces the number of Treg. It also increases the phagocytic activity of macrophages and increases the TNF-α, IL-6, and interleukin 12 (IL-12). In the case of hyperleptinemia, the balance of pro- and anti-inflammatory processes is disturbed and inflammation occurs [58].

In CKD, metabolic degradation in the renal tubules and glomerular filtration is disturbed, which leads to an increase in the concentration of leptin in the blood. The concentration of leptin increases with the progression of the disease [11,59,60]. Korczyńska et al. observed increased expression of leptin in subcutaneous adipose tissue (SAT) among 5th stage CKD pre-dialysis and dialyzed patients [61]. In stages 3–5 of CKD, without dialysis, altered adipokines profile and insulin resistance were associated with VAT, SAT, and intrahepatic fat [62]. Leptin levels are higher in hemodialysis (HD) women than HD men [63].

Leptin influences nutritional status and CVR among the mentioned group of patients. Patients with CKD are at risk of developing PEW, which is associated with a high risk of mortality. The prevalence of PEW among dialyzed patients is up to 80% [64]. One of the mechanisms influencing their occurrence is leptin concentration disturbances. Leptin has catabolic properties such as increasing the metabolic rate and inducing anorexia [65,66]. The secretion of neuropeptides and neurotransmitters involved in the regulation of appetite occurs as a result of Janus Kinase-2 (JAK2) activation caused by the association of leptin with its receptors (ObRb). Leptin increases the secretion of α-melanocyte-stimulating hormone (α-MSH) and cocaine- and amphetamine-regulated transcript (CART), stimulating feeling of satiety, inhibits the synthesis of secretion of one of the strongest appetite stimulants, i.e., neuropeptide Y (NPY) [67,68,69]. The concentration of serum NPY increases, but cerebrospinal fluid (CSF) NPY decreases with the progression of CKD. A low concentration of CSF NPY was associated with cachexia, reduced energy intake, and muscle-wasting among CKD patients [70]. It has been observed that higher serum NPY levels prognosticate cardiovascular mortality among dialysis patients [71,72].

Research has also shown that high levels of leptin among CKD patients are associated with inadequate energy and protein intake, and also with reduced levels of muscle mass [9]. Markaki et al. observed that higher leptin levels in HD and peritoneal dialysis patients were associated with greater fat mass index (FMI) and female gender [73]. On the other hand, other research suggests that patients with PEW have lower leptin levels, which could be related to decreased fat mass. Leptin levels are positively correlated with inflammatory markers, which also affects the risk of malnutrition development [74,75]. More research is needed to understand the mechanism by which leptin affects the occurrence of PEW.

It has been observed that higher serum NPY levels prognosticate cardiovascular mortality among dialysis patients. Leptin contributes to CVD by causing oxidative stress, inflammation, and endothelial cell proliferation [76]. This adipokine increases platelet aggregation and affects the concentration of vascular endothelial growth factor (VEGF), which leads to angiogenesis. Vascular endothelial dysfunction contributes to the development of atherosclerosis, which is the basis of many cardiovascular diseases [60,77]. Furthermore, leptin promotes cardiac hypertrophy by mitogen-activated protein kinase (MAPK) signaling [78].

Lu et al. observed that higher serum leptin levels among patients with CKD were a risk factor of aortic stiffness, measured as the carotid–femoral pulse wave velocity (cfPWV). The study was conducted among 205 patients with CKD stage 3–5 without dialysis and kidney transplantation (KT). Other risk factors of aortic stiffness were higher systolic blood pressure (SBP) and older age [79]. Similar research results were obtained in HD patients and those after KT. In addition, in Kuo et al.’s study, elevated serum leptin level among HD patients was correlated with body mass index (BMI) and fat mass [80,81]. One study found that leptin levels were associated with CVR among HD patients only with a larger waist circumference (>102 cm) [82].

However, low leptin concentration is an independent predictor of mortality among CKD patients. This was confirmed in a study carried out on HD patients and kidney transplant recipients (KTRs). The cohort study involving 1214 KTRs showed that, despite the adverse impact of high leptin level on graft function and severity of inflammation, the risk of mortality was 10% lower for every 10 ng/mL higher leptin concentration [83,84].

### 6.2. Adiponectin

Adiponectin, a 244 amino acid protein, plays an important role in insulin sensitivity by increasing fatty-acid oxidation and reducing gluconeogenesis. Adiponectin has two types of receptors—adipoR1 and adipoR2 [85,86]. The former is found in all tissues and the latter mainly in the liver. The adipoR1 receptors in the excretory system are present in the proximal tubule cells and the glomerulus cells, i.e., in the cells of the endothelium, podocytes, mesangium, and the epithelium of Bowman’s capsule. Adiponectin crosses the glomerular filtration barrier, binds to the adipoR1 receptor on the above-mentioned structures and acts on them by activating the adenosine monophosphate-activated protein kinase (AMPK) pathways [86]. AMPK stimulation induces ATP formation processes, including fatty acid oxidation, which protects against obesity and metabolic disorders, and thus indirectly protects the proper functioning of the kidneys. However, in obesity, the concentration of adiponectin is reduced. Kidneys are exposed to the harmful effects of increased levels of pro-inflammatory adipokines and are devoid of the protective effects of adiponectin. Adiponectin may also play a role in the development of obesity-related albuminuria [86,87,88].

The reduced concentration of adiponectin causes translocation of the zonula occludens (ZO-1) proteins from the podocyte epithelial layer into the cytosol. The ZO-1 proteins are structural tight-junction proteins that are responsible for the tightness of the epithelium and the proper function of slit diaphragms. The slit diaphragm is the structure found between the foot processes of the podocytes and acts as a sieve to prevent high-molecular-weight proteins from entering the urine. The structural proteins of slit diaphragms are nephrin and podocin, which determine the proper functioning of this structure. Additionally, low levels of adiponectin have been associated with low levels of nephrin in the renal cortex, which increases the permeability of the filtration barrier [89]. Adiponectin has opposite actions to leptin. Contrary to the action of leptin, decreased levels of adiponectin result in increased synthesis of TGF-β1. This results in cell hypertrophy and increased collagen synthesis, which causes renal fibrosis [90].

Adiponectin has a protective effect on the vascular endothelium. It inhibits the action of the endothelial transcription factor—nuclear factor-kappa β (NF-κβ), through the activation of AMPK. The reduction in NF-κβ activity in the endothelium inhibits the expression of pro-inflammatory adhesion proteins such as vascular cell adhesion molecule-1 (VCAM-1), E-selectin, and intercellular adhesion molecule-1 (ICAM-1). It causes the adhesion of monocytes to the endothelium of blood vessels, which is a key point in the development of atherosclerosis. On the one hand, this directly affects the renal structures. If the damage to the blood vessels occurs within the kidneys, it leads to a reduction in blood flow and local ischemia of the cells. It causes cell death or damage to their structure and kidney damage. On the other hand, the ongoing inflammatory process further increases renal dysfunction [91].

In addition, the low concentration of adiponectin leads to increased activation of NADPH oxidase 4 (Nox4) and the formation of oxidative stress [92]. Excessive amounts of ROS produced by mitochondria can lead to cellular damage and the progression of renal dysfunction [93].

Adiponectin levels are reduced in obesity, atherosclerosis, and metabolic syndrome. Despite the frequent occurrence of metabolic disorders, patients with CKD treated conservatively have from two to three times higher concentrations of serum adiponectin compared to healthy people. In patients requiring dialysis, the concentrations are even higher [94,95]. After successful KT, adiponectin levels decrease [96].

Adiponectin affects the nutritional status and CVR of patients with CKD. Increased serum adiponectin level is associated with lower BMI, waist circumference, albumin level, female sex, and older age [97,98,99]. Inverse associations were also observed among adiponectin levels and SAT, VAT, total body fat, and lean body mass [100]. Moreover, studies suggest a negative correlation between adiponectin levels and hand-grip [101]. Adiponectin may also affect bone turnover. It has been observed in HD patients that higher levels of adiponectin correlate with a decline in bone mineral density [102]. A high concentration of adiponectin may reflect malnutrition, which indicates a poor prognosis. Hyun et al. assessed the nutritional status and adiponectin concentration in 1303 pre-dialysis patients. Based on regression analysis, higher adiponectin levels were associated with PEW independent of many other factors [103]. In another study conducted among dialysis patients, a positive correlation between adiponectin and Malnutrition Inflammation Score (MIS) was observed, which indicates worsened nutritional status [104,105].

Research results regarding the influence of adiponectin on CVR is inconclusive. It was considered that high levels of adiponectin should benefit the health of people with CKD, which has been confirmed in many studies where lower adiponectin levels were associated with a higher incidence of cardiovascular events [106,107,108]. On the other hand, different studies have observed that high levels of this adipokine are associated with a higher risk of death, including CVR. Hence, it is sometimes called the “adiponectin paradox” [109]. Menon et al. showed that an increase in serum adiponectin among stages 3–4 of CKD by 1 µg/mL was associated with a 6% higher CVR [110]. In a MADRAD study of 501 HD patients, higher adiponectin levels were also associated with a higher risk of mortality. Moreover, the concentration of adiponectin was positively correlated with high-density lipoprotein (HDL) and negatively correlated with total cholesterol and LDL [100]. High levels of adiponectin are also connected with macroalbuminuria, which is an adverse cardiovascular risk factor [111]. A synergistic effect of high adiponectin concentration and low BMI on CVR was observed [112]. The mechanism of the impact of adiponectin on CVR is unknown. Adipokine may increase the occurrence of inflammation, interfere with hematopoiesis, and increase the concentration of atrial natriuretic peptide (ANP) and brain natriuretic peptide (BNP), which are indicators of heart failure [113,114].

It has been established that the Lep/Adpn ratio may be a better indicator of the risk of developing CVD and mortality in CKD patients than the concentration of adiponectin or leptin alone. Lep/Adpn ratio reflects a disturbed function of adipose tissue [115]. Frühbeck et al. proposed the opposite ratio: adiponectin-to-leptin (Adpn/Lep). A low Adpn/Lep ratio was associated with increased inflammation and the occurrence of oxidative stress, which increases the risk of CVD [116].

### 6.3. Zinc-α2-Glycoprotein

Zinc-α2-glycoprotein (ZAG) is a protein secreted by various organs, including AT and renal tubular cells, and is another adipokine that is reduced in obesity. Increased expression of the ZAG gene in AT is caused by glucocorticosteroids and androgens [117]. ZAG regulates the secretion of other adipokines in AT. It reduces the secretion of leptin and increases the secretion of adiponectin. Thus, its low concentration contributes to the persistence of obesity [118]. At physiological concentrations, ZAG increases the expression of peroxisome proliferator-activated receptor γ (PPARγ) and early B-cell factor 2 (EBCF-2) genes by stimulating cyclic AMP (cAMP). This, in turn, initiates an increase in the synthesis of UCP1 and PR/SET domain 16 (Prdm16) proteins, which are involved in the browning process of WAT, which results in increased energy expenditure and leads to lipolysis [119].

ZAG is excreted in the urine due to damage to the basal glomerulus membrane, which may be caused by various glomerulopathies. In diabetic patients, urinary excretion of ZAG increases. It has been suggested that it may be a biomarker of diabetic nephropathy. There is a positive correlation between serum ZAG levels and the value of creatinine and eGFR. Urinary ZAG concentration correlates with the albumin/creatinine ratio [120,121].

Chronic inflammation and elevated leptin levels, which are common in obesity, reduce the secretion of ZAG in AT. Increasing the activity of ZAG in the event of an excessive energy balance may bring health benefits. A negative correlation was observed between ZAG and BMI and the body fat mass in obese subjects [122]. However, in states of catabolism such as cancer cachexia, this adipokine is associated with a deterioration in nutritional status [119].

Patients with CKD have significantly increased plasma levels of ZAG and increased synthesis in WAT compared to the healthy population, which may be associated with impaired renal excretion and the occurrence of uremia [123,124]. Moreover, inflammation and oxidative stress may increase ZAG secretion [125].

ZAG can be a new biomarker of malnutrition due to its lipolytic properties. It activates hormone-dependent lipase (HSL) via cAMP and stimulation of adenylate cyclase [126]. Overexpression of ZAG inhibits lipogenesis by reducing the expression of the genes of enzymes involved in this process, such as fatty-acid synthase (FAS) and acetyl-CoA carboxylase [127]. The serum concentration of ZAG is positively correlated with the serum levels of TG and negatively with HDL cholesterol and albumin levels [128,129].

Obese HD patients have a significantly lower concentration of this adipokine compared with patients with normal body weight [130]. Some studies revealed a negative correlation between the ZAG concentration and the percentage of body fat mass and the sum of skinfolds [131].

Bouchara et al. assessed the effect of ZAG on mortality in HD patients. High ZAG level is a predictor of all-cause mortality and CVR independent of age, or nutritional or metabolic status [126]. In another study, ZAG was negatively associated with TNF-α and VCAM-1, which is a marker of atherosclerosis [131].

### 6.4. Adipose Triglyceride Lipase

Adipose triglyceride lipase (ATGL) is an enzyme encoded by the patatin-like phospholipase domain containing 2 (PNPLA2) gene. It catalyzes the first lipolysis reaction, i.e., the hydrolysis of triacylglyceride (TAG) to diacylglyceride (DAG). ATGL levels are lower in overweight and obese people than in healthy people with normal body weight [132]. Chronic ATGL adipokine deficiency increases the infiltration of immune cells into AT and enhances the inflammatory processes [133]. In terms of kidney function, ATGL deficiency leads to lipid accumulation, damage to the glomerular filtration barrier, and proteinuria. Decreased ATGL levels may increase intracellular ROS formation, which may result in F-actin fiber redistribution, foot-process fusion, and podocyte apoptosis [134]. ATGL gene expression is induced by ZAG in VAT and SAT [135].

As far as it is known, research on the comparison of ATGL concentration in people with normal kidney function and CKD has not been conducted.

Alipoor et al. assessed the concentration of adipokines and nutritional status in HD patients and found that serum ATGL levels were significantly higher in moderate-wasting than in normal-to-mild wasting patients. The increase in ATGL levels was associated with a 21% increase in the severity of wasting. In addition, ATGL concentration was correlated with TG. ATGL levels are positively correlated with FFAs, which indicates that it affects lipolysis [136]. In another study, ATGL levels did not differ between people with normal and excessive body weight, although there was a direct relationship between ATGL levels and the percentage of body fat mass [130].

**Figure 3:**
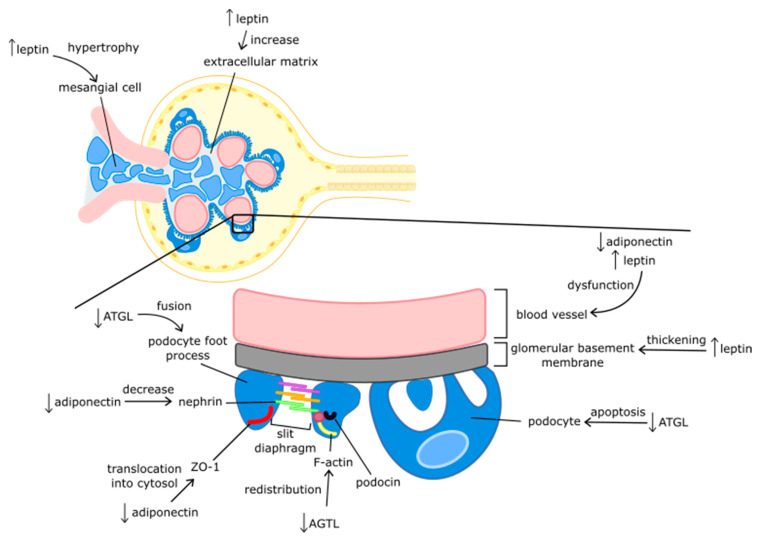
The influence of adipokines on the structures of the kidneys. Abbreviations: ATGL—adipose triglyceride lipase; ZO-1—zonula occludens; ↑—increased levels; ↓—decreased levels.

There are no studies on the effects of ATGL on CVR in patients with CKD. Salatzki et al. showed that in animal models with normal kidney function, the removal of ATGL in AT prevents pressure-induced left ventricular heart failure, which confirms the adverse effect of lipolysis on heart function [137]. However, other studies found an adverse effect of ATGL deficiency on the cardiovascular system. ATGL deficiency has been associated with increased wall thickness and cardiac fibrosis [138,139]. The impact of adipokines on nutritional status and CVR is summarized in Table 1 and on kidney structures in Figure 3.

## 7. Conclusions

Obesity leads to the development of many other diseases through the occurrence of chronic inflammation, oxidative stress, and disorders of adipokine secretion. Obesity and the associated impaired secretion of adipokines contribute to the development of CKD and its progression. However, CKD also influences the concentration of adipokines, nutritional status, and CVR in patients with CKD. There is a mutual influence on the axis of the kidney and AT. Despite the broadening knowledge of obesity and its possible treatment methods, the number of obese people is still increasing. Correspondingly, although there is a lot of research on CKD, many mechanisms are not yet completely understood, for example, the mechanism of the development of nutritional disorders and CVR. In particular, ZAG and ATGL are adipokines that are not sufficiently studied. There is little literature on the concentration of these adipokines concerning the nutritional status and CVR in patients with CKD. Although the manuscript contains information summarizing many publications, not all existing articles on a given topic were considered. Additionally, some of the source texts are reviews and research publications. More research is needed on this topic.

## Figures and Tables

**Table 1:** Profile of adipokines in chronic kidney disease and their influence on nutritional status and cardiovascular risk in CKD.

Activity	Leptin	Adiponectin	ZAG	ATGL
Plasma levels in obesity	↑	↓	↓	↓
Plasma levels in CKD	↑	↑	↑	U
Malnutrition/PEW	↑/↓	↑	↑	↑
Oxidative stress	↑	↑	↑	↑
Inflammation	↑	↑	↑	↑
CVR	↑	↑/↓	↑	U

Abbreviations: ZAG—zinc-alpha2-glycoprotein; ATGL—adipose triglyceride lipase; CKD—chronic kidney disease; PEW—protein energy-wasting; CVR—cardiovascular risk; U—unknown; ↑—increased levels; ↓—decreased levels; ↑/↓—conflicting results.

## Data Availability

Not applicable.
